# Burden of Pruritus in Advanced CKD and Hemodialysis: Results From National Kidney Foundation Surveys

**DOI:** 10.1016/j.xkme.2023.100635

**Published:** 2023-03-25

**Authors:** Dale Lee, Joseph A. Vassalotti, Gail Torres, Linda Singleton-Driscoll

**Affiliations:** 1 Icahn School of Medicine at Mount Sinai, New York, NY; 2 National Kidney Foundation, New York, NY; 3 Chléire Consulting, Inc, Richmond, VA

To the Editor:

People living with chronic kidney disease (CKD), particularly those in the later stages and those treated with hemodialysis, are at an increased risk of CKD-associated pruritus (CKD-aP), an uncomfortable condition that can negatively affect the quality of life and health outcomes.^1^, ^2^, ^3^, ^4^, ^5^, ^6^, ^7^, ^8^, ^9^, ^10^ The itch is often chronic and less is known about pruritus in earlier CKD stages.^1^, ^2^, ^3^, ^4^ The patient-reported effect of pruritus is important across the spectrum of kidney diseases risk stratification.

We conducted 2 similar online surveys of patient experience with pruritus, both using National Kidney Foundation social media to capture US adults, aged 18 years or older, who met the survey criteria. The first survey, conducted from November 11 to November 27, 2020, enrolled patients who were treated with hemodialysis. The second, conducted from June 16 to 21, 2021, focused on patients who self-reported with CKD stage G2, G3, G4, or G5 (Fig S1). Item S1 reports the detailed methods and Item S2 reports the survey instruments that align with the worst itch numeric rating scale.

Among the 1,629 patients who participated in the 2 surveys, 692 patients were receiving hemodialysis and 937 patients with CKD, with a wide range of kidney disease severity (Table 1). The population was broadly distributed across the US census regions, with most being younger than 40 years or younger than the average adult patient with CKD and receiving hemodialysis in the United States.^11^ Most of the patients had some college education and were employed. More than 50% of those receiving hemodialysis had education beyond high school and were employed, which is relatively higher than the US surveillance data.^11^

**Table 1:** Survey Respondent Characteristics

Characteristic	Patients not Receiving Dialysis and With CKD G2 (n = 152)	Patients Not Receiving Dialysis and With CKD G3 (n = 113)	Patients Not Receiving Dialysis and With CKD G4 (n = 258)	Patients Not Receiving Dialysis and With CKD G5 (n = 414)	Patients With CKD and Receiving Hemodialysis (n = 692)
Age, y, n (%)					
<35	108 (71)	87 (77)	114 (44)	182 (44)	252 (36)
35-44	43 (28)	24 (21)	130 (51)	181 (44)	167 (24)
45-54	1 (1)	1 (1)	13 (5)	41 (10)	65 (9)
55-64	0 (0)	0 (0)	0 (0)	5 (1)	31 (5)
≥65	0 (0)	1 (1)	0 (0)	1 (0)	23 (3)
Mean ± SD	31.3 ± 5.3	31.2 ± 6.6	35.2 ± 5.3	36.0 ± 7.1	38.5 ± 11.8
			(1 missing)	(4 missing)	(154 missing)
Female sex, n (%)	67 (44)	60 (53)	139 (54)	236 (57)	245 (35)
			(1 missing)	(3 missing)	(153 missing)
Employment status, n (%)					
Employed or student	136 (90)	108 (96)	221 (86)	299 (73)	402 (58)
	(1 missing)		(2 missing)	(3 missing)	(154 missing)
Race/ethnicity^a^, n (%)					
White	129 (85)	89 (79)	207 (81)	292 (71)	375 (54)
Black	16 (11)	15 (13)	38 (15)	85 (21)	80 (12)
Other	7 (5)	9 (8)	6 (2)	14 (3)	68 (10)
Hispanic	3 (2)	8 (7)	7 (3)	21 (5)	49 (7)
			(3 missing)	(4 missing)	(155 missing)
US Census region, n (%)					
Northeast	13 (9)	11 (10)	34 (13)	27 (7)	63 (9)
Midwest	31 (21)	18 (16)	38 (15)	47 (11)	109 (16)
South	53 (35)	41 (36)	99 (38)	167 (40)	190 (28)
West	54 (36)	43 (38)	75 (29)	161 (39)	165 (24)
	(1 missing)		(12 missing)	(12 missing)	(165 missing)
Education, n (%)					
High school or less	33 (22)	9 (8)	86 (34)	75 (18)	102 (15)
Some college or technical school	54 (36)	41 (36)	57 (22)	173 (42)	216 (31)
College graduate	63 (42)	63 (56)	95 (37)	157 (38)	200 (29)
Postgraduate studies	1 (1)	0 (0)	18 (7)	6 (1)	20 (3)
	(1 missing)		(2 missing)	(3 missing)	(154 missing)
How long been living with kidney disease, n (%)					
≤6 mo	8 (5)	2 (2)	11 (4)	14 (3)	
7-12 mo	22 (14)	9 (8)	89 (34)	77 (19)	
1-2 y	55 (36)	41 (37)	72 (28)	171 (41)	
2-5 y	55 (36)	53 (47)	61 (24)	109 (26)	
>5 y	12 (8)	7 (6)	25 (10)	42 (10)	
		(1 missing)		(1 missing)	
How long been on dialysis, n (%)					
≤6 mo					88 (13)
7-12 mo					191 (28)
1-2 y					155 (22)
2-5 y					159 (23)
>5 y					99 (14)

Abbreviations: CKD, chronic kidney disease; n, number; SD, standard deviation.

a May exceed 100% owing to multiple responses.

In these surveys, ≥87% of the patients with CKD, across the stages of kidney disease or receiving hemodialysis, reported experiencing pruritus (Fig 1). Approximately half of the patients had itchy skin for a year or longer (43%-61%), and some reported being itchy most, if not all, of the time (range across the kidney disease populations, 12%-42%).

**Figure 1:**
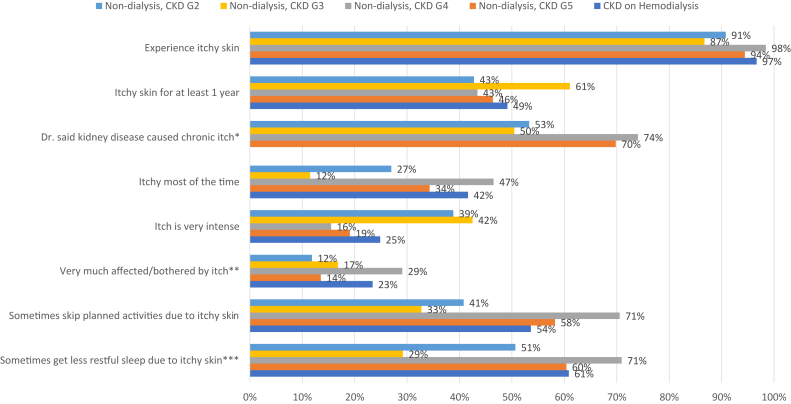
Survey results of pruritus effects. ∗Question not asked of patients receiving hemodialysis. ∗∗Patients treated with dialysis rated how bothersome they found an occurrence of itchy skin; Patients not treated with dialysis rated how much itchy skin affected them. ∗∗∗Patients treated with dialysis rated how often they got less restful sleep owing to itchy skin; Patients not treated with dialysis rated how often they had trouble sleeping through the night owing to itchy skin. CKD, chronic kidney disease.

Approximately half of the patients with CKD G2 or G3 reported their doctor informing them that their pruritus was caused by kidney disease, increasing to 70%-74% among patients with CKD G4 or G5.

Patients with CKD G2 or G3 are particularly likely to report that the itch was very intense (39%-42%) compared with patients with both CKD G4 or G5 (16%-19%) and receiving hemodialysis (25%). Many patients with CKD reported being very much affected/bothered by itchy skin (range across the CKD stages, 12%-29%). Similarly, patients treated with hemodialysis reported being very much affected/bothered by itchy skin (23%).

Patients described many ways that itchy skin affected them. One example is skipping planned activities because of itchy skin. Patients with CKD G4 or G5 (58%-71%) or those treated with hemodialysis (54%) reported this more often than patients with CKD G2 or G3 (33%-41%). Many patients also reported that the itchy skin sometimes caused sleep problems. More than half of the patients with CKD G4 or G5 (60%-71%) reported that itchy skin interferes with sleeping through the night, and a similar percentage was noted among those who were treated with hemodialysis (61%).

This cross-sectional snapshot of the experiences of the US adults suggests that pruritus is a common, chronic symptom in patients with CKD and treated with hemodialysis. When mild, it is uncomfortable and can lead to skin excoriations. However, when severe, sleep and social functioning are affected. This study has several limitations. First, the survey population was self-selected and nonrepresentative. Given that our data were recorded from online surveys, the graphs may not accurately represent the whole population of those who have late-stage CKD and those on hemodialysis. In addition, transplant respondents were potentially included in the CKD survey. Our survey likely had a selection bias for younger, better educated, and mostly employed people who tend to use social media. In the adult population, data are conflicting on whether younger or older patients are more likely to report pruritus.^9^ The authors also speculate that awareness and education about kidney diseases are likely to be higher among individuals who follow National Kidney Foundation social media. Second, we used a nonvalidated survey tool. Finally, the likely etiology of pruritus in CKD G4, G5, and hemodialysis is uremia, whereas the etiology of pruritus in CKD G2 and G3 is an etiology other than uremia. In CKD G2 and G3, a thorough search of dermatologic, hepatobiliary, hematologic, endocrinologic, and neurologic causes is prudent. Investigation to elucidate the pathogenesis of CKD-aP is ongoing. Histamine and parathyroid hormone have been investigated, but studies have not demonstrated a causal relationship. Recently, the literature has focused on an imbalance of μ-opioid and κ-opioid receptor activation with μ-receptor overactivation as a specific hypothesis in CKD-aP.^10^

Our study highlights the importance of detecting pruritus in CKD and hemodialysis. Treatment should address the primary etiology and symptoms. Pruritus is commonly associated with CKD and hemodialysis with frequent chronicity and a significant negative effect on the quality of life.

## Supplementary Material

Supplementary File (pdf) Figure S1; Item S1; Item S2.
