# Supplementary material for: Burden of Pruritus in Advanced CKD and Hemodialysis: Results From National Kidney Foundation Surveys
Source: Kidney Med. 2023 Mar 25;5(6):100635. doi: 10.1016/j.xkme.2023.100635 (PMC10248877; doi:10.1016/j.xkme.2023.100635)
Supplement: Supplementary File (pdf) — Figure S1; Item S1; Item S2. [file mmc1.pdf]

Figure S1. Flow diagram of recruitment and participation for the two patient studies

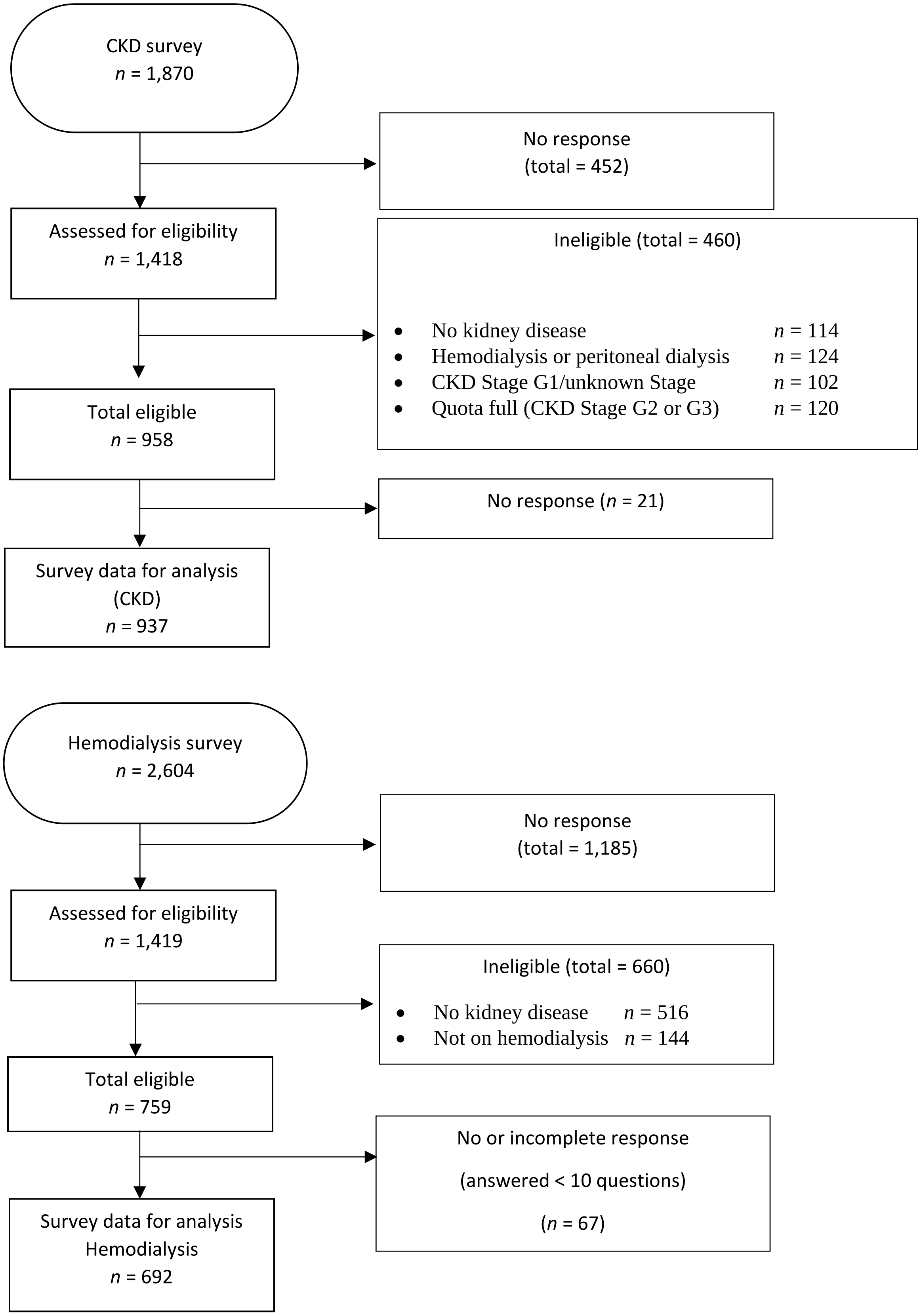

Item S1. Detailed methods

CKD Patient Survey

QUALIFICATIONS: This survey enrolled U.S. adults with CKD stage G2 or higher kidney disease who are not currently undergoing dialysis.

OVERSAMPLE: In order to ensure that we gathered input from at least 300 patients with CKD stage G4 or G5, we closed the study to patients who reported their CKD was at an earlier stage after we had received 265 completed responses from patients who reported their CKD was either Stage G2 or G3. These 265 represent 28% of the 937 responses collected.

SOURCE: The respondents for this study came from the followers of the National Kidney Foundation (NKF) social media. On June 16, 2021, NKF posted on the Facebook page a link to the survey, inviting participation. The first response was received shortly thereafter and responses continued to come in over the following days. To find the remaining CKD stage G4 and G5 patients we sought, alerts about the survey, with the inclusion of a mention of an Amazon.com gift card for $5 (for the first 300 completed responses), were sent via Twitter and LinkedIn on June 22. All responses sought for this study were received by the end of the day.

PROCESS: Clicking on the social media link brought potential respondents to the online survey, where they were asked a series of questions to determine their eligibility to participate, with no indication of the desired qualifications other than kidney disease. Before they could access the survey, patients completed a Completely Automated Public Turing authentication to tell Computers and Humans Apart (CAPTCHA) and subsequent access was restricted to prevent any attempt to load the survey a second time. At the conclusion of the study, the electronic gift cards were emailed by Amazon.com with a thank you note from NKF.

Survey of Hemodialysis Patients

QUALIFICATIONS: This survey enrolled U.S. adults treated with hemodialysis.

SOURCE: The respondents for this study came from the followers of NKF social media. On November 11, 2020, NKF posted on the Facebook page a link to the survey, inviting participation. The first response was received shortly thereafter and responses continued to come in over the following days. The invitation was reposted on November 27, 2020, with the inclusion of a mention of an Amazon.com gift card for $5 (for the first 300 completed responses). All responses sought for this study were received by the end of the day.

PROCESS: Clicking on the social media link brought potential respondents to the online survey, where they were asked a series of questions to determine their eligibility to participate, with no indication of the desired qualifications other than kidney disease. Before they could access the survey, patients completed a CAPTCHA and subsequent access was restricted to prevent any attempt to load the survey a second time. At the conclusion of the study, the electronic gift cards were emailed by Amazon.com with a thank you note from NKF.

Informed consent

There was no research ethics committee nor IRB involvement. We followed "best practices" for surveying patients for their opinion and perception/recollection of their experiences. The survey opening page was seen by each potential respondent that stated the length of the survey, survey topic ("health"), how the data would be used ("to help improve the health of people with kidney disease"), and assured anonymity (which was maintained at all times). Respondents had to hit the "continue" button to begin the survey and after each question, essentially consenting to continue the survey after every question response. If respondents were uncomfortable answering any question after reading it, they could close their browser and end the survey. The survey did not ask for the respondent's name or any other personally identifying information, nor provide a place for them to include unique identifiers other than an email address. Respondents did need to provide an email address if they wanted to receive the gift card, they could enter a "throw-away" email address (it did not have to be their primary email address nor even one used for any other purpose). The email addresses were not kept or stored.

Item S2. Survey instruments

CKD Patient Survey

NON-DIALYSIS CKD PATIENT VIEWS ON PRURITUS

Study Background:

Methodology: Online
Population: Adults with CKD stages G2-5 who are not on dialysis
Target: 300 complete responses with stages G4 or G5 and 200 complete responses with stages G2 or G3
Length: 7-10 minutes

Online Invitation:

To be posted by NKF on social media.

> Do you have CKD? If so, your experiences may be just what we need to help improve the lives of everyone who lives with kidney disease.
>
> Please click here and answer these important questions. We greatly appreciate your time and feedback. The first 300 who complete the 10-minute survey will be given a $5.00 online gift card.

Header for each page:

NATIONAL KIDNEY FOUNDATION.

Labels:

Internet tab (top of page): NKF Health Study
Headings and notes in red will NOT be included in the final, online version
None of the numbers associated with any of the responses will be visible

Opening page:

NATIONAL KIDNEY FOUNDATION.

Welcome to our 10-minute Health Survey!

Your opinions and experience will help us improve the health of people with kidney disease. Your answers will be anonymous and not linked to you.

Hints:

If you skip a question, the next screen will highlight any unanswered question(s) in red and give you the chance to answer it.

Maximizing the size of your browser window will give you the best view of the survey questions.

To go to the first question, simply click the [CONTINUE] button below using your mouse.

Qualifying

Q1. To make sure we ask you the right questions, has your doctor ever told you that you have any of the following? *(check all that apply)* {rotate all but last 2}

Kidney disease/failure → If NOT checked, skip to Disqualified
Heart disease
Diabetes
High blood pressure
High cholesterol
None of these
Prefer not to answer

Q2. What stage is your kidney disease? *(circle or check the one best answer)*

[1] Urinary tract infection → Skip to Disqualified
Chronic Kidney Disease (CKD) Stage 1 → Skip to Disqualified
Chronic Kidney Disease (CKD) Stage 2
Chronic Kidney Disease (CKD) Stage 3
Chronic Kidney Disease (CKD) Stage 4
Chronic Kidney Disease (CKD) Stage 5
Chronic Kidney Disease (CKD), unsure what Stage → Skip to Disqualified
Not sure I have Chronic Kidney Disease → Skip to Disqualified

Q3. Are you currently undergoing: *(circle or check the one best answer)*

[1] Hemodialysis → Skip to Disqualified
[2] Peritoneal Dialysis → Skip to Disqualified
[3] Not currently undergoing dialysis

Experience with Praritus

Q4. How long have you been living with kidney disease?

[1] 6 months or less
[2] 7 – 12 months

[3] 1 – 2 years
[4] 2 – 5 years
[5] More than 5 years
[6] No idea when my kidney disease was diagnosed

Q5. How often do you experience itchy skin?

[1] All of the time
[2] Most of the time
[3] Sometimes
[4] Rarely
[5] Never → Skip to Q16

Q6. Does your itch:

[1] Happen nearly every day
[2] Recur, but not every day
[3] Neither of these → Skip to Q16

Q7. How long ago did you start experiencing itchy skin?

[1] 6 months or less
[2] 7 – 12 months
[3] 1 – 2 years
[4] 2 – 5 years
[5] More than 5 years
[6] Not sure

Q8. What did your doctor tell you is the cause of your chronic itch? {rotate all but last 3}

[1] Skin disease (such as Eczema or hives)
[2] Kidney disease
[3] Liver disease
[4] Other cause (please specify: ________________________)
[5] My doctor didn't say what is causing my chronic itch
[6] Don't recall the cause

Q9. How intense would you say the itch typically is?

[1] Extremely intense
[2] Very intense
[3] Somewhat intense
[4] A little intense

[5] Not at all intense
[6] Varies greatly

Q10. How often do you scratch your itchy skin? ***(check all that apply)***

[1] Most of the time throughout the day
[2] Some of the time throughout the day
[3] A little during the day
[4] Most of the time throughout the night
[5] Some of the time throughout the night
[6] A little during the night
[7] Varies greatly

Q11. Over time has the itch:

[1] Gotten a lot worse
Gotten a little worse
Stayed about the same
Gotten a little better
Gotten a lot better

Q12. Please indicate the intensity of the WORST ITCHING you experienced over the past 24 hours by marking the number that best describes it.

0 (No itch)	1	2	3	4	5	6	7	8	9	10 (Worst itching imaginable)
○	○	○	○	○	○	○	○	○	○	○

Q13. Where on your body do you experience itching? ***(check all that apply)***

[1] Head/scalp
Face
Neck
Shoulder
Chest/front torso
Belly
Arm
Hand
Upper back

Lower back
Groin
Buttocks
Thigh
Calf
Foot
No place

Q14. Do you experience itch mostly on:

[1] Both sides of the body
Only one side of the body
Varies greatly

Q15. Do the places on your body that itch generally:

[1] Stay the same → Skip to 0
Change → Skip to 0

Q16. {ASK if answered 5 to Q5 or 3 to Q6} Suppose you were to develop chronic itchy skin. How much do you think experiencing chronic itchy skin would affect you?

[1] Not at all
[2] A little
[3] Somewhat
[4] Very much
[5] Extremely
[6] Absolutely no idea

Q17. {ASK if answered 5 to Q5 or 3 to Q6} Which of these treatments would you try for itchy skin? *(check all that apply)* {rotate all but last 3}

[1] An over-the-counter skin cream or lotion
[2] A prescription skin cream or lotion
[3] An over-the-counter oral medication
[4] A prescription oral medication
[5] A phosphate binder
[6] Change my diet to lower my phosphorus level
[7] None
[8] No idea

[9] Other (please specify):____

{SKIP to Q24 IF answered Q17}

Q18. In general, how much does your itchy skin affect you?

[1] Not at all
[2] A little
[3] Somewhat
[4] Very much
[5] Extremely

Q19. Which of these treatments have you tried in the last 6 months for itchy skin? *(check all that apply)* {rotate all but last 2}

[1] An over-the-counter skin cream or lotion
[2] A prescription skin cream or lotion
[3] An over-the-counter oral medication
[4] A prescription oral medication
[5] A phosphate binder
[6] Change my diet to lower my phosphorus level
[7] None → Skip to Q22
[8] Other (please specify):____

Q20. Overall, how satisfied are you with how well the treatments control your itch?

[1] Very satisfied
[2] Moderately satisfied
[3] Slightly satisfied
[4] Not at all satisfied

Q21. {Ask if answer 1, 2, 3, 4, or 5 to Q19How effective do you feel the medications you use control the itch?

[1] Very effective
[2] Moderately effective
[3] Slightly effective
[4] Not at all effective

Impact of Pruritus

Q22. How often does itchy skin cause you to: {rotate list}

	Always	Most of the time	Some of the time	Rarely	Never
a) Have trouble falling asleep	[5]	[4]	[3]	[2]	[1]
b) Have trouble sleeping through the night	[5]	[4]	[3]	[2]	[1]
c) Socialize less	[5]	[4]	[3]	[2]	[1]
d) Get less done than you would like	[5]	[4]	[3]	[2]	[1]
e) Have less energy	[5]	[4]	[3]	[2]	[1]
f) Skip planned activities	[5]	[4]	[3]	[2]	[1]
g) Change your routine	[5]	[4]	[3]	[2]	[1]
h) Eat less	[5]	[4]	[3]	[2]	[1]
i) Be in a bad mood	[5]	[4]	[3]	[2]	[1]
j) Feel unhappy or depressed	[5]	[4]	[3]	[2]	[1]
k) Feel like you are losing your mind	[5]	[4]	[3]	[2]	[1]
l) Scratch until skin breaks or bleeds	[5]	[4]	[3]	[2]	[1]

Q23. What are your biggest challenges in dealing with itchy skin? *(check all that apply)* {rotate all but last 2}

[1] My doctor/provider never discusses itching
[2] Itching interferes with my daily activities
[3] Medications I have tried do not control my itch
[4] Medications I have tried give me side effects
[5] My itchy skin is not challenging to me
[6] Other (please specify):____

Q24. What would you like your doctor to know about the challenges of living with kidney disease (and any other conditions you may have)? *(Add as much or as little as you like. You can skip the question (or put 0) if there is nothing you think they should know.)*

____

____

Demographics

Finally a little more about you—so we can look at your answers in combination with those of others

Q25. Gender:

[1] Female
[2] Male
[3] Other/Non-binary

Q26. In what year were you born? ____________________

Q27. Your state? _________________

Q28. Are you typically:

[1] Employed full-time
[2] Employed part-time
[3] Student
[4] Homemaker
[5] Retired
[6] None of these

Q29. Your last year or grade of school completed:

[1] Some high school or less
[2] High school graduate
[3] Some college or technical school
[4] College graduate
[5] Post-graduate studies or degree

Q30. What race or ethnic group do you consider yourself? ***(check all that apply)***

[1] Hispanic/Latinx
[2] Black/African American
[3] White/Caucasian
[4] Native American/American Indian
[5] Asian
[6] Other race/ethnic group_______________
[7] Prefer not to answer

Q31. Is your eGFR (estimated Glomerular Filtration Rate) about: ***(circle or check the one best answer)***

[1] Less than 15
[2] 15 - 29
[3] 30 - 44
[4] 45 - 59
[5] 60 - 89
[6] 90 or greater
[7] No idea

Q32. ***Thank you*** *for taking time to complete this survey and helping improve the health of others**!*** We would like to show our appreciation by emailing the first 300 who complete the survey a $5 Amazon.com Gift Card.*

The email address where I want to receive my $5 Amazon.com Gift Card: ____
The contact information provided here will not be used or shared for any other purpose without your specific expressed consent.

*Amazon.com is not a sponsor of this promotion. Except as required by law, Amazon.com Gift Cards ("GCs") cannot be transferred for value or redeemed for cash. GCs may be used only for purchases of eligible goods at Amazon.com or certain of its affiliated websites. For complete terms and conditions, see www.amazon.com/gc-legal. GCs are issued by ACI Gift Cards, Inc., a Washington corporation. All Amazon ® ,™ & © are IP of Amazon.com, Inc. or its affiliates. No expiration date or service fees.

Disqualified (only shown to those who did not meet the study qualifications)

Thank you for trying to participate in this study.
Unfortunately, we have already received enough responses from people with your qualifications. Wishing you good health!

Survey of ESKD Patients

PATIENT VIEWS ON PRURITUS

Study Background:

Methodology: Online

Population: Adults on hemodialysis
Target: 300 completes
Length: 7-10 minutes

Online Invitation:

To be posted by NKF on social media.

Are you currently undergoing **dialysis** to treat your CKD? If so, your experiences and opinions could help improve the lives of everyone who lives with kidney disease.

Please click here and answer these important questions. We greatly appreciate your time and feedback. The first 300 who complete the 10-minute survey will be given a $5.00 online gift card.

Header for each page:

NATIONAL KIDNEY FOUNDATION.

Labels:

Internet tab (top of page): NKF Health Study
Headings and notes in red will NOT be included in the final, online version
None of the numbers associated with any of the responses will be visible

Opening page:

NATIONAL KIDNEY FOUNDATION.

Welcome to our 10-minute Health Survey!

Your opinions and experience will help us improve the health of people with kidney disease. Your answers will be anonymous and not linked to you.

Hints:

If you skip a question, the next screen will highlight any unanswered question(s) in red and give you the chance to answer it.

Maximizing the size of your browser window will give you the best view of the survey questions.

To go to the first question, simply click the [CONTINUE] button below using your mouse.

Qualifying

Q1. To make sure we ask you the right questions, has your doctor ever told you that you have any of the following? *(check all that apply)* {rotate all but last 2}

[1] Kidney disease/failure → If NOT checked, skip to Disqualified

[2] Heart disease

[3] Diabetes

[4] High blood pressure

[5] Pruritus

[6] High cholesterol

[7] None of these

[8] Prefer not to answer

Q2. Are you currently undergoing: *(circle or check the one best answer)*

[1] Peritoneal Dialysis → Skip to Disqualified

[2] Hemodialysis (at a center)

[3] Hemodialysis (at home)

[4] Not currently undergoing dialysis → Skip to Disqualified

Q3. How long in total have you been on dialysis?

[1] 6 months or less

[2] 7 – 12 months

[3] 1 – 2 years

[4] 2 – 5 years

[5] More than 5 years

[6] Have never been on kidney dialysis → Skip to Disqualified

Awareness & Approach

Q4. How often do you experience itchy skin?

[1] All of the time

[2] Most of the time

[3] Some of the time

[4] A little of the time

[5] Never → Skip to Q10

Q5. How long have you had itchy skin?

[1] 6 months or less

[2] 7 – 12 months
[3] 1 – 2 years
[4] 2 – 5 years
[5] More than 5 years
[6] Not sure

Q6. In the last four weeks, how often did you cut a dialysis session short because of your itchy skin? {presented in reverse order}

[1] Never
[2] 1 - 2 times per month
[3] 1 – 2 times each week
[4] Every session or almost every session

Q7. How intense would you say the itch typically is?

[1] Extremely intense
[2] Very intense
[3] Somewhat intense
[4] A little intense
[5] Not at all intense
[6] Varies greatly

Q8. How bothersome do {"would" replaces "do" if answered 5 to Q4} you find an occurrence of itchy skin?

[1] Not at all bothersome
[2] No more bothersome than anything else I face
[3] A little bothersome
[4] Somewhat bothersome
[5] Very bothersome
[6] Extremely bothersome

Q9. How familiar are you with the name pruritus for itchy skin?

[1] Familiar
[2] I think I have heard of it but didn't know that's what it meant
[3] I have never heard of it
[4] Don't recall

Q10. Who do {"would" replaces "do" and "if" replaces "when" if answered 5 to Q4} you talk with when you experience itchy skin? *(check all that apply)* {rotate all but last 2}

[1] Friend/family member
[2] Pharmacist
[3] Nephrologist/kidney doctor
[4] Doctor other than a nephrologist (such as primary care or dermatologist)
[5] Nurse
[6] Dialysis technician
[7] Dietitian
[8] Social worker
[9] No one
[10] Other (who?) ______________________________

Q11. What kinds of healthcare providers have given you information about pruritus or itchy skin? *(check all that apply)* {rotate all but last 2}

[1] Nephrologist
[2] Primary care physician
[3] Dermatologist
[4] Pharmacist
[5] Nurse
[6] Dialysis technician
[7] Dietitian
[8] Social worker
[9] None of my healthcare providers→ Skip to Q19
[10] Some other healthcare provider (who?):_________________________

Q12. What did the healthcare professional recommend you do to address your pruritus or itchy skin? *(check all that apply)* {rotate all but last 3}

[1] Change my diet
[2] Drink more water
[3] Take one or more prescription drugs to help the itch
[4] Take an over-the-counter remedy/supplement/herb or home remedy to help the itch
[5] Take phosphate binders or change phosphate binder prescription
[6] Don't scratch the itch

[7] Apply more lotion
[8] Visit a dietitian
[9] Visit a specialist
[10] None of these, although I have talked with my provider about my itchy skin
[11] Never talked to a provider about my itchy skin
[12] Other, please specify________________________________

Q13. Which of these have you tried so far for itchy skin? ***(check all that apply)*** {rotate all but last 2}

[1] Change my diet
[2] Drink more water
[3] Take one or more prescription drugs for pruritus/itchy skin → Ask Q14
[4] Take an over-the-counter remedy/supplement/herb or home remedy → Ask Q14
[5] Take phosphate binders or change phosphate binder prescription
[6] Don't scratch the itch
[7] Apply more lotion
[8] Visit a dietitian
[9] Visit a specialist
[10] None of these
[11] Never have itchy skin → Skip to Q21

Q14. {Ask if answer 3 or 4 to Q19}Which medications or remedies have you tried to control your pruritus or itchy skin? ***(check all that apply)*** {rotate all but last two}

[1] Topical emollients (such as Eucerin)
[2] Topical steroid
[3] Non-steroid medicated topical preparation
[4] Antihistamine (such as Benadryl)
[5] Gabapentin/pregabalin (such as Neurontin or Lyrica)
[6] UV light therapy
[7] None
[8] Other (what?____)

Q15. When it comes to managing itchy skin, what one strategy is the: {rotate all but last one}

	**most** effective	**least** effective
a) Change my diet	1	1
b) Drink more water	2	2
c) Take one or more prescription drugs for itchy skin	3	3
d) Take an over-the-counter remedy/supplement/herb or home remedy	4	4
e) Take phosphate binders or change phosphate binder prescription	5	5
f) Don't scratch the itch	6	6
g) Apply more lotion	7	7
h) Visit a dietitian	8	8
i) Visit a specialist	9	9
j) Other (what?_____________)	10	10

Q16. Overall, how satisfied are you with how well the treatments are able to control your itch?

[1] Very satisfied
[2] Somewhat satisfied
[3] A little satisfied
[4] Not at all satisfied

Q17. How often does itchy skin cause you to: {rotate list}

	Always	Most of the time	Some of the time	Rarely	Never
a) Get less restful sleep	[5]	[4]	[3]	[2]	[1]
b) Socialize less	[5]	[4]	[3]	[2]	[1]
c) Get less done than you would like	[5]	[4]	[3]	[2]	[1]
d) Have less energy	[5]	[4]	[3]	[2]	[1]
e) Skip planned activities	[5]	[4]	[3]	[2]	[1]
f) Change your routine	[5]	[4]	[3]	[2]	[1]
g) Be in a bad mood	[5]	[4]	[3]	[2]	[1]
h) Feel unhappy or depressed	[5]	[4]	[3]	[2]	[1]
i) Feel like you are losing your mind	[5]	[4]	[3]	[2]	[1]
j) Miss a dialysis session	[5]	[4]	[3]	[2]	[1]
k) End a dialysis session early	[5]	[4]	[3]	[2]	[1]
l) Scratch until skin breaks or bleeds	[5]	[4]	[3]	[2]	[1]

Q18. What is your biggest challenge in dealing with itchy skin?

____

____

Barriers

Q19. How often do you encounter these barriers in managing your pruritus? {rotate list}

	Always	Most of the time	Some of the time	Rarely	Never
a) Lack of communication between doctors/members of my medical team	[5]	[4]	[3]	[2]	[1]
b) Access to treatment	[5]	[4]	[3]	[2]	[1]
c) Cost of treatment	[5]	[4]	[3]	[2]	[1]
d) Side effects or reactions to medications	[5]	[4]	[3]	[2]	[1]
e) Insufficient disease control with treatment	[5]	[4]	[3]	[2]	[1]
f) Inadequate education on my current conditions and how to manage them	[5]	[4]	[3]	[2]	[1]
g) Difficulty following all recommended care and treatment	[5]	[4]	[3]	[2]	[1]
h) Constraints on provider support	[5]	[4]	[3]	[2]	[1]
i) Limitation of available treatments	[5]	[4]	[3]	[2]	[1]
j) Inadequate education on related illnesses I might contract	[5]	[4]	[3]	[2]	[1]
k) Providers lack adequate knowledge on causes and treatment of itchy skin	[5]	[4]	[3]	[2]	[1]

Q20. Please list any other barriers you encounter.

____

Communication Preferences

Q21. How do you like to learn about treatment options and issues? *(check all that apply)* {rotate all but last 1}

a) Video/DVD	1
b) Webinar	2
c) Teaching card	3
d) Information sheet	4
e) Printed booklet/brochure	5
f) E-newsletter	6
g) Internet/Social Media	7
h) Smartphone app	8
i) Infographic	9
j) Information helpline	10
k) Support group	11
l) One-on-one with a doctor	12
m) One-on-one with a nurse	13
n) One-on-one with a social worker	14
o) One-on-one with a dietitian	15
p) One-on-one with another patient	16
q) None of these	17

Q22. Where do you most like to turn to find health information, such as when you face a health challenge?

____

____

Q23. What type of information is important to you when faced with a new health challenge such as itchy skin? {rotate list}

		Essential	Very helpful	Somewhat helpful	Not at all helpful
a)	Description of disease or issue	[4]	[3]	[2]	[1]
b)	Self-care tips	[4]	[3]	[2]	[1]
c)	Risk factors	[4]	[3]	[2]	[1]
d)	Ways to reduce risk	[4]	[3]	[2]	[1]
e)	Treatment options	[4]	[3]	[2]	[1]
f)	Importance of treatment	[4]	[3]	[2]	[1]
g)	What issues are important to report	[4]	[3]	[2]	[1]
h)	Where to find more information	[4]	[3]	[2]	[1]

Q24. What type of information have healthcare providers mentioned to you about pruritus or itchy skin? *(check all that apply)* {rotate all but last 2}

[1] Description of disease
[2] Self-care tips
[3] Risk factors
[4] Ways to reduce risk
[5] Treatment options
[6] Importance of treatment
[7] Importance of reporting it to your healthcare team
[8] Where to find more information
[9] None of these
[10] Other, please specify:____

Demographics

Finally a little more about you—so we can look at your answers in combination with those of others

Q25. Gender:

[1] Female
[2] Male
[3] Other/Non-binary

Q26. In what year were you born? ____________________

Q27. Your state? _________________

Q28. Are you typically:

[1] Employed full-time

[2] Employed part-time

[3] Student

[4] Homemaker

[5] Retired

[6] None of these

Q29. Your last year or grade of school completed:

[1] Some high school or less

[2] High school graduate

[3] Some college or technical school

[4] College graduate

[5] Post-graduate studies or degree

Q30. What race or ethnic group do you consider yourself? ***(check all that apply)***

[1] Hispanic/Latinx

[2] Black/African American

[3] White/Caucasian

[4] Native American/American Indian

[5] Asian

[6] Other race/ethnic group______________

[7] Prefer not to answer

Q31. ***Thank you*** *for taking time to complete this survey and helping improve the health of others!* We would like to show our appreciation by emailing the first 300 who complete the survey a $5 Amazon.com Gift Card.*

The email address where I want to receive my $5 Amazon.com Gift Card: ____

The contact information provided here will not be used or shared for any other purpose without your specific expressed consent.

*Amazon.com is not a sponsor of this promotion. Except as required by law, Amazon.com Gift Cards ("GCs") cannot be transferred for value or redeemed for cash. GCs may be used only for purchases of eligible goods at Amazon.com or certain of its affiliated websites. For complete terms and conditions, see www.amazon.com/gc-legal. GCs are issued by ACI Gift Cards, Inc., a Washington corporation. All Amazon ® ,™ & © are IP of Amazon.com, Inc. or its affiliates. No expiration date or service fees.

Disqualified (only shown to those who did not meet the study qualifications)

Thank you for trying to participate in this study.
Unfortunately, we have already received enough responses from people with your qualifications. Wishing you good health!
